# Cytokines in treated and untreated Pompe patients

**DOI:** 10.1186/1471-2474-14-S2-P6

**Published:** 2013-05-29

**Authors:** N Karabul, S Gökce, M Kirchner, W Mannhardt, E Mengel

**Affiliations:** 1 Center for Pediatric and Adolescent Medicine, University Medical Center, Mainz, Germany

## Introduction

Glycogen storage disease type II (Pompe disease or acid maltase deficiency) is an autosomal recessive metabolic disorder caused by a deficiency of the lysosomal enzyme acid alpha-glucosidase. Accumulation of glycogen in the lysosomes damages muscle cells throughout the body. In response to that damage, we hypothesized that cytokines (a family of proteins that mediate innate and adaptive immunity) would be elevated. We investigated 30 (15 female, 15 male) Pompe patients before ERT start and 1 year after ERT and used high-resolution ELISA.

## Results

Before ERT start in 20 patients (12f, 8m) we saw elevated TNFy and Interleukin-2. After treatment more than 50% TNFy was normalized. But after treatment some cytokines were elevated. We don’t see any correlation between antibody levels against ERT in these patients.

## Conclusion

Our results showed that some patients had elevated cytokines and monocytes. Our hypothesis is that also immunological factors play also a role in disease, causing the development of pain in Pompe disease. Now we are looking for correlations between pain and cytokines levels, and results will follow.

